# Artificial Intelligence in Cryo-Electron Microscopy

**DOI:** 10.3390/life12081267

**Published:** 2022-08-19

**Authors:** Jeong Min Chung, Clarissa L. Durie, Jinseok Lee

**Affiliations:** 1 Department of Biotechnology, The Catholic University of Korea, Bucheon-si 14662, Gyeonggi, Korea; 2 Department of Biochemistry, University of Missouri, Columbia, MO 65211, USA; 3 Department of Biomedical Engineering, Kyung Hee University, Yongin-si 17104, Gyeonggi, Korea

**Keywords:** artificial intelligence, deep learning, neural network, cryo-electron microscopy

## Abstract

Cryo-electron microscopy (cryo-EM) has become an unrivaled tool for determining the structure of macromolecular complexes. The biological function of macromolecular complexes is inextricably tied to the flexibility of these complexes. Single particle cryo-EM can reveal the conformational heterogeneity of a biochemically pure sample, leading to well-founded mechanistic hypotheses about the roles these complexes play in biology. However, the processing of increasingly large, complex datasets using traditional data processing strategies is exceedingly expensive in both user time and computational resources. Current innovations in data processing capitalize on artificial intelligence (AI) to improve the efficiency of data analysis and validation. Here, we review new tools that use AI to automate the data analysis steps of particle picking, 3D map reconstruction, and local resolution determination. We discuss how the application of AI moves the field forward, and what obstacles remain. We also introduce potential future applications of AI to use cryo-EM in understanding protein communities in cells.

## 1. Introduction

In cells, macromolecules perform their biological roles in the context of complex networks, exchanging binding partners and altering assembly states [1]. This molecular network can be determined by the structural analysis of macromolecules, which is crucial for understanding functional mechanisms and designing new drugs to exploit those functions in disease states [2]. Since 2013, we have experienced a boom of structural discovery with respect to biomolecules using cryo-electron microscopy (cryo-EM), now arguably the most powerful tool in structural biology. Distinct from other conventional structural approaches, such as X-ray crystallography (XRC) and Nuclear Magnetic Resonance (NMR), cryo-EM can characterize the intact forms of various biomolecules and their interacting partners in sizes ranging from small individual proteins to large complexes, at near-atomic resolution [3,4]. This “resolution revolution” was achieved through advancements in both instrumental hardware (e.g., vitrification machines, electron sources, and direct electron detectors [5,6,7]) and image processing software (e.g., Relion [8], CryoSPARC [9], Cistem [10], Scipion [11]). Single-particle analysis (SPA) cryo-EM uses a large number of extracted particles randomly oriented in vitreous ice, resulting in a unique advantage in studying macromolecular complexes. Using this technique, it is possible to observe numerous conformational variations between states and understand the flexibility of disordered regions of biomolecular complexes [12,13].

**Figure 1:**
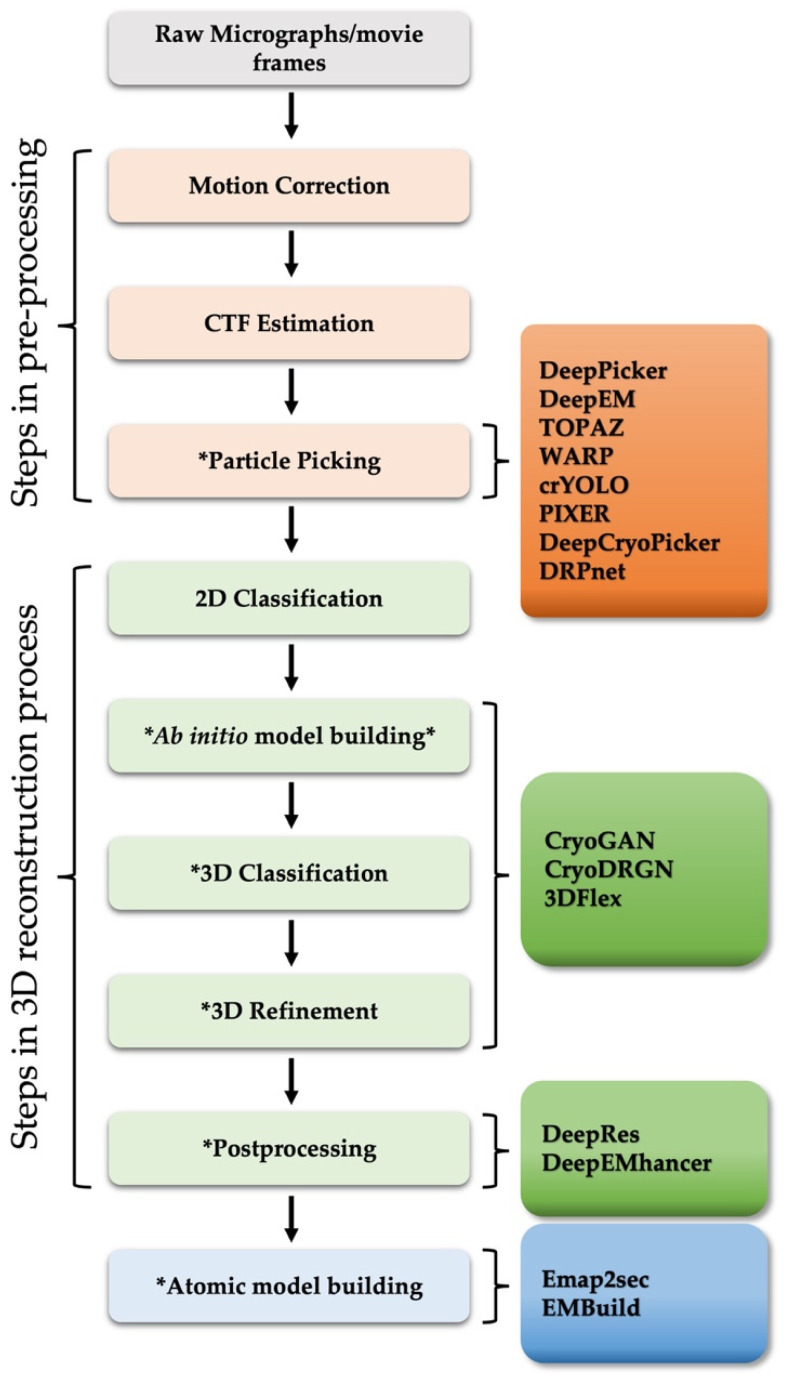
General workflow diagram of SPA 3D reconstruction. The asterisk (*) represents steps in which deep learning algorithms have been actively applied recently. The remaining steps, including motion correction, CTF estimation and 2D classification, were not discussed in this paper. For more details, please refer to [32,33,34]. The deep learning-based approaches (right boxes) introduced in this review were placed at each corresponding stage of the computational pipeline.

Structural analysis using cryo-EM of biologically important macromolecules is increasingly complex, scaling with the size of the complexes studied and the size of the datasets collected by better, faster cameras. The heterogeneity of dynamic macromolecules can be a great barrier to reconstructing high-resolution 3D structures. To address the challenge of structural heterogeneity, semi-automated image processing algorithms and pipelines have been developed [9,11]. However, despite computational developments, a high level of user skill, time, and attention are still required, which not only limits processing speed, but also introduces significant variability to the end result [14,15]. With recent advances in artificial intelligence (AI) technology, several fully automated deep learning-based image processing approaches have been applied to the workflow of cryo-EM 3D reconstruction and atomic structure determination, including steps such as particle picking [16,17,18,19,20,21,22,23], 3D map reconstruction [24,25,26], resolution determination [27,28], map sharpening [29], and model building [30,31], all relying on neural networks that are trained on “big data” (Figure 1 and Table 1).

This review paper will introduce various neural network-based programs actively developed and currently used in the cryo-EM, explaining how the new programs address current gaps in the field, and propose methodological areas for further development.

## 2. Pre-Processing: Particle Picking

One of the most important tasks in SPA cryo-EM for high-resolution 3D reconstruction, second only to preparing optimal samples, is particle selection. During data collection, a low electron dose is used to minimize radiation damage to biological samples, which generates noisy images with low contrast, making it difficult to recognize particles from raw micrographs [35]. For this reason, the reconstruction of a reliable 3D map requires a sufficiently large set of particle images, the selection of which is the first bottleneck in the image processing steps of SPA. Over the past decades, many particle recognition methods have been proposed, most of which are based on template matching, edge detection and feature extraction [36,37,38]. Template matching is the most popular particle selection approach. Template matching uses the cross-correlation of patched micrographs with calculated particle image templates [39]. This semi-automated particle selection method performs well with “good quality images”, meaning those with strong signal-to-noise ratio (SNR) and a good contrast. However, because this method depends greatly on the quality of the micrographs, its performance is significantly reduced for heterogeneous samples commonly found with biological macromolecules. The weakness of requiring exemplary micrographs for dynamic, non-ideal biochemical samples also applies to other conventional approaches (e.g., edge detection and feature extraction). If semi-automated particle selection is not reliable, users resort to manual particle selection, which requires a great deal of time and effort and introduces many opportunities for user error or bias.

Over the past few years, new automated particle selection methods have been introduced (e.g., DeepPicker [16], DeepEM [20]) using deep learning algorithms and convolutional techniques to extract features from massive quantities of data through layers in neural networks [40]. A convolutional neural network (CNN) is a biological process-inspired deep learning algorithm that differentiates one from the other by accepting input images and assigning importance (weight and bias) to various aspects of the images [41]. Similarly to multi-layer perceptions, each convolution layer is connected within the network; that is, the values of one layer act as inputs of the next layer, so that the algorithm learns complex patterns [17].

DeepPicker [16] and DeepEM [20] are some of the earliest models of fully automated particle recognition tools. These particle selecting tools crop micrographs with a default step size by a sliding window and generate several image patches, which are then the input of CNN that classifies the extracted patches into positive (actual particle) or negative (background noise) images. For training purpose, DeepEM requires hundreds of manually selected particles [20], while DeepPicker has an alternative training scheme which uses a pretrained network with similarly shaped molecules as training data for particle picking [16]. These neural network-based particle picking tools have contributed significantly to particle selection from challenging datasets. However, these approaches still have some drawbacks. The computational costs can be high, as these programs generate several image patches by a sliding window to crop each collected micrograph and grouping these patches into “good” or “bad” [17,18,21]. In addition, these approaches are not suitable for large particles or samples with ice contamination.

Recently, several advanced deep learning-based particle picking packages have been released to address the above issues, such as TOPAZ [22], WARP [23], and crYOLO [17]. TOPAZ, one of the most popular particle-picking tools in recent years, uses a similar deep learning system to DeepPicker. In contrast to existing deep learning-based particle selection tools, including DeepPicker and DeepEM, TOPAZ uses a relatively small number of training samples, accomplished by using unlabeled samples in place of negative samples [22]. Since negative data corresponding to non-particles have more diverse characteristics, the manual selection of non-particles is an important but tedious task. To overcome this challenge, TOPAZ formulates the problem description as a positive-unlabeled learning problem, which trains a model given a small number of positive samples and the remaining samples, which are unlabeled, are understood to be non-particles. WARP employs a deep residual network (ResNet) architecture [42], which allows for the training of deeper CNNs by effectively skipping some connections or layers [23]. WARP was trained with real data from the electron microscopy public image archive (EMPIAR) [43] and synthetic data from the protein data bank (PDB) [44]. In addition, WARP corrects micrograph motion and estimates the local defocus, which ultimately identifies high-contrast artifacts and provides accurate particle picking results. crYOLO utilizes an object detection approach called “You Only Look Once” (YOLO) [45], which is a state-of-the-art approach in deep neural network in terms of both speed and accuracy. The advantage of crYOLO is that it requires only a single pass of the full image instead of multiple passes of cropped regions [17]. Moreover, as crYOLO uses a single pass of the full image, it is more appropriate for detecting the larger context around a particle of interest [17]. These advanced, fully automated, deep learning approaches appear to be more suited for cryo-EM image processing than the conventional methods. Nonetheless, as the size of cryo-EM datasets dramatically increases without the improvement of SNR, the field must pay more attention to the issue of low-SNR images, which significantly reduce the detection accuracy of these advanced particle picking tools.

## 3. Three-Dimensional (3D) Map Reconstruction

SPA cryo-EM is a method that determines the 3D structure of macromolecules at the atomic level by imaging many individual particles, isolated from a biological sample, frozen in a cryogenic state [46]. Although it deals with biological substances, 3D reconstruction is an astonishing product of physical and mathematical theories. Since it is not the main topic of this review paper, the detailed theories describing 3D reconstruction will not be covered, but we will briefly introduce some basic principles used in SPA cryo-EM.

Transmission electron microscopy (TEM) produces 2D projection images, so the basic concept of SPA 3D reconstruction is to generate a 3D model by computationally combining various 2D images representing different orientations/views of the biological sample [47]. The most important part of this process is to accurately estimate the orientation and translational shift (pose) of each individual particle image extracted from the raw micrograph. However, as mentioned in the previous section, cryo-EM collects images using a very low electron dose to protect the sample from radiation damage, which results in a low SNR thus hindering accurate pose estimation. To improve the SNR, several particle images are collected and aligned in the same orientation through several computational approaches [48]. There are various strategies for pose estimation that have been implemented in EM image processing software packages. One approach is projection matching [49], in which unknown poses of each experimental image are assigned by comparing the unknown pose with a computationally produced initial 3D reference model. Although the projection matching approach is relatively simple, the accuracy of pose estimation is significantly reduced at lower SNR, so projection matching requires high computational costs [34]. In the advancement of 3D reconstruction algorithms in SPA cryo-EM, some approaches using statistical weighting of projection images have been introduced, including maximum-likelihood (ML) approaches [50,51]. In a ML implementation, each individual particle image is not directly assigned a single pose (the best match). Instead, each particle image is given a set of probable of orientations and similarity scores which eventually are used as weights in 3D reconstruction [34,36,47,50,51]. During each iteration, the estimation scores are improved until meeting a convergence criterion [51,52]. However, as it is still difficult to search all possible 3D maps, results heavily depend on the first estimate of the initial 3D model, resulting in an artifact known as model-bias [34]. More recently, an implementation with ab-initio (initial model-free) model using a stochastic gradient descent (SGD) algorithm has been proposed to address the model-bias derived misassignment issue [9]. Although SGD minimizes optimization problems in SPA 3D reconstruction, it is not sufficient for high-resolution 3D reconstruction (further refinement steps are required) and is not a complete initial model-free approach as an initial guess for modeling is still needed [51]. There are still many improvements needed, but SGD has recently received a lot of attention because it greatly advances the application of the deep learning field to cryoEM [53,54].

### 3.1. Model Building, 3D Classification, and 3D Refinement

The molecular mechanisms of proteins, protein complexes, and other biological macromolecules are essential for maintaining life. Until recently, however, these molecular mechanisms have been largely inferred from static 3D structures. Now, if we can analyze the presence and distribution of different conformations related to the versatile roles these macromolecules play, a more complete understanding of the secrets of life can be achieved.

SPA cryo-EM is considered an optimal approach for determining high-resolution 3D structures of a variety of macromolecules, especially for heterogenous, flexible, and/or dynamic complexes [55,56]. The predominant approach used for heterogenous reconstruction in SPA image processing packages is a discrete classification, such as 3D classification or heterogeneous refinement (e.g., local refinement [10,36] and multi-body refinement [57]), in which each particle can only belong to one class or pose, or to another. There is no information about possible relationships between classes. For such an approach, it is necessary to specify an initial model to provide information on the underlying structural state, which can result in a fatal errors or biases as described above [24]. The most problematic point is that the 3D reconstruction approach is not suitable for observing various conformations of complexes that undergo continuous structural changes [24]. More recently, a linear subspace model (Principal Component Analysis, PCA [58,59]), called 3D Variability Analysis (3DVA) [60], has been proposed to resolve the continuous distribution of related conformations of macromolecules. One caveat, however, is that 3DVA may introduce artifacts when the structural change is incorrectly approximated by linear interpolations through underlying volumes [24,60].

In SPA cryo-EM, utilization of deep learning algorithms is mostly limited to pre-processing steps, including raw image denoising [61] and particle selection [16,17,18,19,20,21]. Very recently, a few neural network-based approaches, which are feature unsupervised learning and no requirement for prior training, have been applied to determine SPA 3D reconstructions, including CryoGAN [25], CryoDRGN [24], and 3D Flexible Refinement (3DFlex) [26]. CryoGAN [25] modifies generative adversarial networks (GANs) [62], in which the generator network of a classical GAN is replaced by a cryo-EM Physics Simulator, to reconstruct a 3D model for the continuous variability of biomolecules. CryoDRGN [24] uses a modified variational auto-encoder (VAE) [63], called amortized variational inference approach, for the posterior estimation of the volume, while 3DFlex [26] adopts an auto-decoder model performing direct inference to increase the accuracy of the posterior estimation of the conformational coordinates. Although the auto-decoder approach enables the reconstruction of flexible regions with higher resolution to understand more detailed information about the dynamics of macromolecules, it requires more computational resources than the encoder-based approaches [26].

### 3.2. Postprocessing

Along with the remarkable advancements in equipment and image processing programs, high-resolution 3D EM maps of various complexes using SPA cryo-EM have reached the point where detailed information about the biomolecules can be revealed. For this reason, resolution evaluation and verification of reconstructed 3D maps is increasingly important. Despite this importance, the concept of resolution has not yet been completely defined in the electron microscopy field, and the various approaches currently used have yet to be fully agreed upon [64,65]. Currently, the most commonly used approach to define the resolution of the 3D EM map is based on Fourier Shell Correlation (FSC) curves, calculating the correlations of different resolutions of Fourier space at a given threshold between two independent 3D maps of the same molecule [66,67]. However, this approach remains somewhat controversial for a few reasons. First, this approach requires setting a reference threshold for the measured information [27]. Second, the assessment of the 3D reconstruction through this approach is not sensitive to isotropic filtering of the whole dataset and may vary depending on the local features of the density map [27,28].

One of the first local resolution approaches to evaluate the quality of local regions of an EM map is BlocRes [64], which estimates the resolution of local regions based on FSC through a sliding window over the entire density map. In addition to the limitations of the FSC described above, this approach has the additional disadvantage of having to specify the size of the moving window [27,64]. Another recently developed approach is ResMap [65] which estimates the local resolution by detecting a 3D sinusoidal wave above the noise level for each point on a density map. More recently, MonoRes [68] has been proposed to define the local resolution of a 3D electron density map. This most recent approach is based on a similar principle to the ResMap method, but it uses monogenic amplitude at different frequencies. This MonoRes approach estimates the local resolution by comparing the monogenic signals with the corresponding monogenic amplitude of the noise within a defined resolution range. However, all approaches, including recently proposed methods, require significant computational processing time, and additional estimation of noise variance, so the final estimates produced by various approaches differ considerably [28].

With the emergence of deep learning algorithms as new technology in the cryo-EM field, some neural network-based approaches have also been proposed for local resolution estimation procedures [27,28]. Among them, the recently released CNN-based automatic local resolution estimation method, called DeepRes [27], addresses some of the drawbacks of the conventional approaches that are currently being used in cryo-EM. In particular, it is possible to detect local changes in the quality of 3D EM maps caused by various post-processing procedures, such as isotropic filtering, model/non-model-based local sharpening, and noise suppression, which are frequently used in the course of a modeling workflow [27]. As there is no universally accepted approach to determine local resolution estimation yet, and various debates continue, further development and research of deep learning-based methods for this application are necessary.

## 4. Atomic Model Building

**Figure 2:**
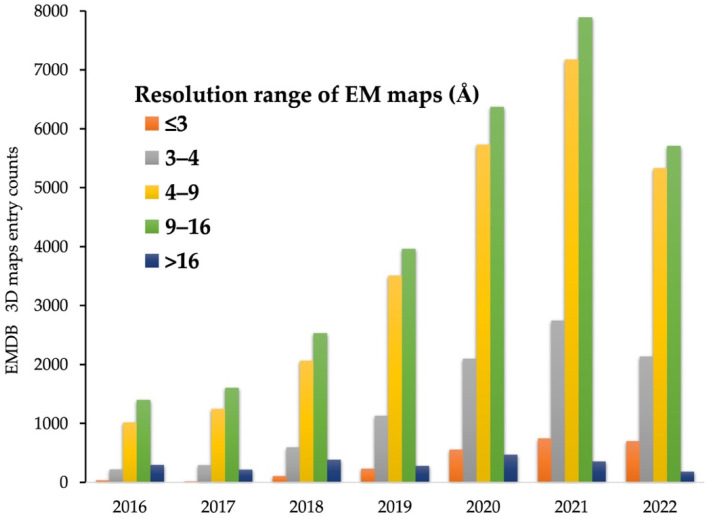
Electron Microscopy Data Bank (EMDB) [69] entry counts at given resolution ranges over the last 7 years.

As a result of recent innovative technological advances in cryo-EM instrumentation and analysis tools, including those described in this review, structural analysis of important biological systems that were previously intractable has become possible. The broadening diversity of analytic approaches is fueling sensational innovation that can reveal the secrets of biology at a molecular level, with wide-ranging impacts on human health and our understanding of the world around us. In fact, the number of high-resolution maps obtained by cryo-EM is rapidly increasing (Figure 2) [69], and in recent years, it is approaching the number of 3D models reconstructed through XRC, which has long been the standard of structural techniques [44,69,70]. However, the goal of structural analysis is not simply to reconstruct a 3D map in atomic detail, but to understand what those atomic structures suggest about molecular mechanisms such as interactions between biological macromolecules. In this regard, there is still much room for growth.

High-resolution (<3 Å) EM maps are now sufficient for determining high-quality atomic structures using only slightly modified software [71,72] originally designed for XRC. In addition, *de novo* atomic model building using 3D maps with near-atomic resolution (around 4 Å), which accounts for the greatest portion of cryo-EM maps deposited currently, is now commonplace [73,74,75]. However, many 3D EM maps are still reconstructed at intermediate resolutions, ranging from ~5–8 Å (Figure 2), due to inherent properties of macromolecular complexes (e.g., high flexibility and multiple conformational states) which have a significant impact on high-resolution 3D reconstruction, and these intermediate resolution maps are insufficient for determining atomic structures [30,69,76]. Moreover, with the recent increase in the number of intermediate resolution maps obtained through sub-tomogram averages of cryo-electron tomography (cryo-ET), the most rapidly developing method in the cryo-EM field, accurate structural determination approaches for intermediate EM maps are urgently needed [31,77,78,79]. Indeed, as of 2022, only about 2000 out of 4000 maps with intermediate resolution (in the range of ~5–8 Å) deposited to EMDB, have a complete atomic structure [44,69].

Efforts are underway to solve this urgent issue, and several reliable approaches are currently being explored. One common approach is to model the atomic structure by fitting a given template, such as a previously determined homologous atomic structure or a predicted structure based on amino acid sequences, to an EM map through a series of refinement processes [80,81,82,83]. However, many problems remain with these approaches to atomic model building using low-resolution EM maps. The success of the model fitting approach described above requires a high degree of user expertise, so the final model is heavily influenced by the skill and experience of the individual user performing the fitting and refinements. Model fitting for protein complexes with flexible regions and various conformational states requires complicated procedures which inevitably introduces errors in the final model, while also incurring high computational costs [31]. In addition, the software used in most approaches was primarily designed for single-chain protein fitting, but we now are often investigating macromolecular complexes made up of multiple proteins or proteins and nucleic acids. Thus, EM map segmentation for each subunit of the complex must be performed before full-scale model fitting [30,31]. For accurate map segmentation, the information contained in the EM map is crucial, but low-resolution EM maps carry less information than high-resolution maps, increasing the challenge of building a reliable complete structure through model fitting approaches for an intermediate resolution EM map, especially for molecular complexes [31].

To address such challenges, some deep learning-based approaches have been proposed to automatically build the atomic structure from relatively low-resolution EM maps, including Emap2sec [30] and EMBuild [31]. Emap2sec has implemented a CNN-based algorithm which has the advantage of performing local structure detection across the whole 3D map [30]. Its performance was benchmarked using various EM maps with intermediate resolution ranging from 5 Å to 10 Å, resulting in more accurate detection of secondary structure with improved validation scores in the resulting 3D maps compared to those obtained using traditional approaches [71,84]. However, despite showing improved detection accuracy, an observed limitation is in building the specific secondary structure, such as alpha-helices and beta-strands into the detected local regions [30]. More recently, another deep learning-based approach (called EMBuild) using a nested U-net (UNet++) [85], a fully convolution network (FCN) which is a more powerful architecture for image segmentation, has been applied to atomic model building from intermediate resolution EM maps [31]. EMBuild was evaluated not only on SPA EM maps (4–8 Å), but also maps obtained by sub-tomogram averaging (4–9 Å), and it showed excellent performance in building reliable atomic structures into intermediate resolution 3D maps [31]. Although many modifications are still needed in the future, recently developed deep learning-based algorithms are expected to serve as an essential tool for simplifying structural determination in intermediate resolution cryo-EM maps.

## 5. Future Applications

Through the era of the “resolution revolution” of cryo-EM, brought about by advances in instrumentation and sample preparation, we have learned a tremendous amount of structural and functional information about numerous, important macromolecular complexes. However, due to the inherent characteristics of macromolecular complexes that perform various roles in cells (e.g., heterogeneity), structural analysis becomes more complicated compared to analysis of individual proteins, and many challenges are amplified in these higher quality data sets [13]. To overcome these problems that cannot be solved by existing structural analytic methods, several approaches have been introduced building on the advancements of deep learning-based algorithms. In particular, CNN-based models excel in image classification and particle recognition steps, which are the most fundamental steps in the cryo-EM image processing workflow [86]. Moreover, some neural-network algorithms capable of reconstructing high-resolution 3D structures for heterogenous samples are also proposed [24,25,52]. More recently, deep learning-based approaches related to post-processing, the final step associated with the enhancement of the reconstructed 3D electron density map, have also been launched [29]. Thus, we are in the early stages of a period of rapid advancement in data analysis capabilities built on the strength of AI and machine learning. This advancement will open a new frontier in the types of samples accessible to high resolution characterization by cryoEM.

In SPA cryo-EM, scientists typically analyze the structures and functions of various molecular complexes which have been isolated and taken out of their original context in cells. These purified samples are used in in vitro experiments to understand their important physiological processes. However, this approach does not capture the characteristics of molecular complexes in cells, especially versatile protein complexes that function through interactions with several functionally synchronized partners in their original environments, called protein communities [87,88].

The next frontier in analyzing these protein communities is to directly observe and structurally characterize native cell extracts. A recent study combined electron microscopy and mass spectrometry data to visualize each protein complex within the communities in native cell extracts [89]. In addition, with the recent advancements in cryo-EM [3], it is now possible to obtain high-resolution data on native cell extracts, and many related studies have been published recently [13,88,90,91]. However, despite these advances, the high complexity of cell extracts makes it difficult to properly quantify and 3D reconstruct molecular complexes interacting within the cell extract [92]. To meet this need, neural network-based approaches are being developed to effectively detect and isolate in silico particles of different shapes and sizes within protein communities from EM images of cell extracts [13]. The biggest obstacle to this strategy is how effectively we can determine the 3D model of each component from heterogeneous 2D projections of imaged cell extracts [92]. Advances in recently published AI-based protein structure prediction tools [93,94,95] have opened a new path for the study of these cell extracts. Researchers will now be able to easily access reliable model prediction tools, gaining insight into the 3D structure of molecular complexes applicable to the study of native cell extracts.

## Figures and Tables

**Table 1:** Recently introduced deep learning-based cryo-EM image processing approaches.

Name	Application Area	Reference
DeepPicker	Particle Recognition	Wang et al., 2016 [16]
DeepEM	Particle Recognition	Zhu et al., 2017 [20]
TOPAZ	Particle Recognition	Bepler et al., 2019 [22]
WARP	Particle Recognition	Tegunov et al., 2019 [23]
crYOLO	Particle Recognition	Wagner et al., 2019 [17]
PIXER	Particle Recognition	Zhang et al., 2019 [21]
DeepCryoPicker	Particle Recognition	Al-Azzawi et al., 2020 [19]
DRPnet	Particle Recognition	Nguyen et al., 2021 [18]
CryoGAN	3D Reconstruction	Gupta et al., 2021 [25]
CryoDRGN	3D Reconstruction	Zhong et al., 2021 [24]
3DFlex	3D Reconstruction	Punjani et al., 2021 [26]
DeepRes	Local resolution	Ramirez-Aportela et al., 2019 [27]
DeepEMhancer	Map Sharpening	Sanchez-Garcia et al., 2021 [29]
Emap2sec	Model building	Maddhuri Venkata Subramaniya et al., 2019 [30]
EMBuild	Model building	He et al., 2022 [31]
